# Supplementary material for: Spatially resolved analysis of Pseudomonas aeruginosa biofilm proteomes measured by laser ablation sample transfer
Source: PLoS One. 2021 Jul 22;16(7):e0250911. doi: 10.1371/journal.pone.0250911 (PMC8297752; doi:10.1371/journal.pone.0250911)
Supplement: S1 File — S1 Fig depicts the strategy for exposing anaerobic surface of a biofilm. S1 Table lists the P. aeruginosa proteins identified from three-day biofilms grown on polycarbonate membranes. S2 Table lists the proteins identified from axial ablations on transwell membrane insert grown biofilms. (PDF) [file pone.0250911.s001.pdf]

Supporting Information for

Spatially resolved analysis of *Pseudomonas aeruginosa* biofilm proteomes measured by laser ablation sample transfer

Aruni Chathurya Pulukkody,[1] Yeni P. Yung,[1] Fabrizio Donnarumma,[2] Kermit K. Murray[2], Ross P. Carlson,[3] and Luke Hanley[1,*]

[1]Department of Chemistry, University of Illinois at Chicago, Chicago, IL 60607, USA

[2]Department of Chemistry, Louisiana State University, 232 Choppin Hall, Baton Rouge, LA 70803, USA

[3]Department of Chemical and Biological Engineering, Center for Biofilm Engineering, Montana State University, Bozeman, MT 59717

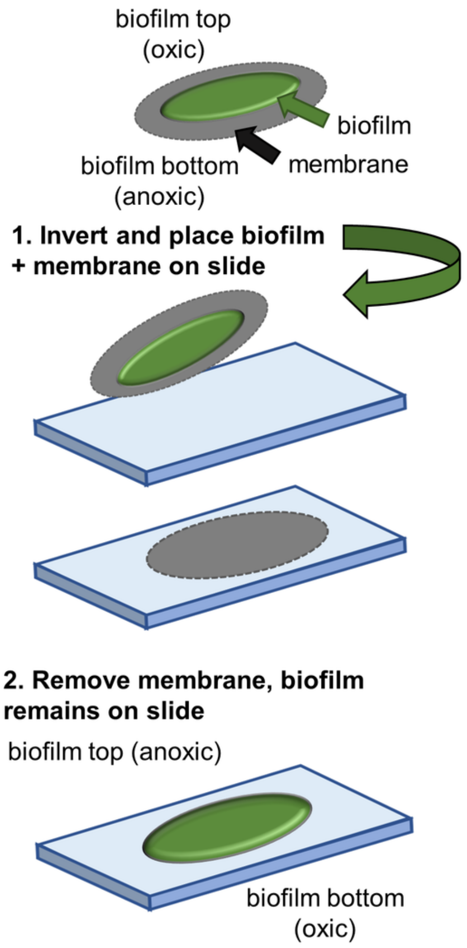

S1 Fig. Strategy for exposing anaerobic surface of a biofilm. Process involved freezing the membrane on dry ice and inversion on a glass slide. The membrane was peeled off the glass slide to expose the anoxic side of the biofilm.

S1 Table: ***P. aeruginosa* proteins identified from three-day biofilms grown on polycarbonate membranes.** Proteins in brown font correspond to those that appear only in older cell region (1 mm square, brown region in Fig 5A) while proteins in green font appear only in newer cell region of biofilm (2 mm square, green region in Fig 5A). Black proteins are common to both regions. All reported proteins displayed at least two unique peptides and ≥10% sequence coverage. Data is preliminary as it was collected from only one pooled LC-MS/MS analysis each, so no statistical analysis was possible.

Protein	KEGG ID	Description of Protein	Unique Peptides	Sequence Coverage (%)
DapA	PA1010	Dihydrodipicolinate synthase	2	10
RplS	PA3742	50S ribosomal protein L19	2	31
RibH	PA4053	6,7-dimethyl-8-ribityllumazine synthase	2	15
RplN	PA4253	50S ribosomal protein L14	5	40
RplV	PA4258	50S ribosomal protein L22	2	28
RpsJ	PA4264	30S ribosomal protein S10	2	23
RpsL	PA4268	30S ribosomal protein S12	2	15
AtpA	PA5556	ATP synthase alpha chain	2	34
MetK	PA0546	Methionine adenosyltransferase	8	26
Pgk	PA0552	Phosphoglycerate kinase	5	16
TufA;TufB	PA4265	Elongation factor Tu	12	64
RplK	PA4274	50S ribosomal protein L11	7	57
RplA	PA4273	50S ribosomal protein L1	9	50
RplJ	PA4272	50S ribosomal protein L10	5	39
RpsG	PA4267	30S ribosomal protein S7	6	37
RplC	PA4263	50S ribosomal protein L3	5	27
RplD	PA4262	50S ribosomal protein L4	5	24
RplW	PA4261	50S ribosomal protein L23	2	19
RplB	PA4260	50S ribosomal protein L2	5	21
RpsC	PA4257	30S ribosomal protein S3	6	40
RplP	PA4256	50S ribosomal protein L16	3	23
RplX	PA4252	50S ribosomal protein L24	3	36
RpsN	PA4250	30S ribosomal protein S14	2	22
RpsH	PA4249	30S ribosomal protein S8	3	25
RplR	PA4247	50S ribosomal protein L18	5	31
RpsE	PA4246	30S ribosomal protein S5	5	41
RplO	PA4244	50S ribosomal protein L15	6	45
RpsK	PA4240	30S ribosomal protein S11	3	24

RpoA	PA4238	DNA-directed RNA polymerase alpha chain	8	23
HemL	PA3977	Glutamate-1-semialdehyde 2,1-aminomutase	4	16
Ndk	PA3807	Nucleoside diphosphate kinase	4	28
RlmN	PA3806	Dual-specificity RNA methyltransferase RlmN	3	12
RplS	PA3742	50S ribosomal protein L19	4	50
Tsf	PA3655	Elongation factor Ts	7	25
PyrG	PA3637	CTP synthase	6	12
Eno	PA3635	Enolase	4	13
RecA	PA3617	Protein RecA	8	30
FadB	PA3014	Fatty acid oxidation complex subunit alpha	7	15
SthA	PA2991	Soluble pyridine nucleotide transhydrogenase	7	24
Efp	PA2851	Elongation factor P	3	22
RplT	PA2741	50S ribosomal protein L20	4	24
NuoA2	PA2637	NADH-quinone oxidoreductase subunit A 2	2	20
ClpX	PA1802	ATP-dependent Clp protease ATP-binding subunit ClpX	4	11
FabA	PA1610	3-hydroxydecanoyl-[acyl-carrier-protein] dehydratase	2	12
SucC	PA1588	Succinate--CoA ligase [ADP-forming] subunit beta	10	53
ProS	PA0956	Proline--tRNA ligase	6	12
PhhB	PA0871	Pterin-4-alpha-carbinolamine dehydratase	4	36
GroES	PA4386	10 kDa chaperonin	4	50
LpxC	PA4406	UDP-3-O-acyl-N-acetylglucosamine deacetylase	7	27
RpsI	PA4432	30S ribosomal protein S9	5	37
MurA	PA4450	UDP-N-acetylglucosamine 1-carboxyvinyltransferase	6	17
MscL	PA4614	Large-conductance mechanosensitive channel	2	16
IlvC	PA4694	Ketol-acid reductoisomerase (NADP(+))	9	34
InfB	PA4744	Translation initiation factor IF-2	5	11
DapB	PA4759	4-hydroxy-tetrahydrodipicolinate reductase	2	11
PurH	PA4854	Bifunctional purine biosynthesis protein PurH	6	13

RplI	PA4932	50S ribosomal protein L9	4	32
RpsF	PA4935	30S ribosomal protein S6	4	27
PurA	PA4938	Adenylosuccinate synthetase	4	11
HslU	PA5054	ATP-dependent protease ATPase subunit HslU	6	13
SecB	PA5128	Protein-export protein SecB	2	10
PckA	PA5192	Phosphoenolpyruvate carboxykinase (ATP)	7	16
RpmB	PA5316	50S ribosomal protein L28	3	40
AtpC	PA5553	ATP synthase epsilon chain	3	25
AtpD	PA5554	ATP synthase subunit beta	21	63
AtpG	PA5555	ATP synthase gamma chain	4	19
AtpA	PA5556	ATP synthase subunit alpha	4	38
YidC	PA5568	Membrane protein insertase YidC	6	13
PepA	PA3831	Cytosol aminopeptidase	9	24
IscS	PA3814	Cysteine desulfurase IscS	4	11
GuaA	PA3769	GMP synthase [glutamine-hydrolyzing]	7	15
AspS	PA0963	Aspartate--tRNA(Asp/Asn) ligase	7	22
AstB	PA0899	N-succinylarginine dihydrolase	6	17
GroEL	PA4385	60 kDa chaperonin	28	48
SecA	PA4403	Protein translocase subunit SecA	14	18
Eco	PA2755	Ecotin	2	12
GatA	PA4483	Glutamyl-tRNA(Gln) amidotransferase subunit A	6	15
RplU	PA4568	50S ribosomal protein L21	8	70
RplY	PA4671	50S ribosomal protein L25	7	56
GlyS	PA0008	Glycine--tRNA ligase beta subunit	8	13
ThrS	PA2744	Threonine--tRNA ligase	7	11
Pnp	PA4740	Polyribonucleotide nucleotidyltransferase	23	37
GlmM	PA4749	Phosphoglucosamine mutase	4	10
DnaJ	PA4760	Chaperone protein DnaJ	7	31
CoaD	PA0363	Phosphopantetheine adenylyltransferase	3	23
ArgS	PA5051	Arginine--tRNA ligase	6	11
HutU	PA5100	Urocanate hydratase	8	17
AhcY	PA0432	Adenosylhomocysteinase	5	13
LptD	PA0595	LPS-assembly protein LptD	2	12
RpoB	PA4270	DNA-directed RNA polymerase subunit beta	40	35

RplE	PA4251	50S ribosomal protein L5	6	37
RpsM	PA4241	30S ribosomal protein S13	7	52
RpsD	PA4239	30S ribosomal protein S4	6	32
RpsB	PA3656	30S ribosomal protein S2	6	18
RdgC	PA3263	Recombination-associated protein RdgC	3	12
SerC	PA3167	Phosphoserine aminotransferase	6	19
Tig	PA1800	Trigger factor	14	42
CysS	PA1795	Cysteine--tRNA ligase	4	11
PctA	PA4309	Methyl-accepting chemotaxis protein PctA	3	24
DapD	PA3666	2,3,4,5-tetrahydropyridine-2,6-dicarboxylate N-succinyltransferase	4	13
AruC	PA0895	Succinylornithine transaminase/acetylornithine aminotransferase	6	21
EstA	PA5112	Esterase EstA	6	13
KatA	PA4236	Catalase	9	25
Zwf	PA3183	Glucose-6-phosphate 1-dehydrogenase	8	23
RatA	PA4767	Ribosome association toxin RatA	2	13
LysC	PA0904	Aspartokinase	5	14
HupB	PA1804	DNA-binding protein HU-beta	2	32
ArcB	PA5172	Ornithine carbamoyltransferase, catabolic	12	31
OprI	PA2853	Major outer membrane lipoprotein	4	39
OprF	PA1777	Outer membrane porin F	14	41
ArcA	PA5171	Arginine deiminase	13	35
GltA	PA1580	Citrate synthase	9	16
BraC	PA1074	Leucine-, isoleucine-, valine-, threonine-, and alanine-binding protein	12	42
FliC	PA1092	B-type flagellin	16	46
BraF	PA1071	High-affinity branched-chain amino acid transport protein BraF	3	15
Gor	PA2025	Glutathione reductase	4	12
AlgC	PA5322	Phosphomannomutase/phosphoglucomutase	9	24
Gap	PA3195	Glyceraldehyde-3-phosphate dehydrogenase	9	36
Edd	PA3194	Phosphogluconate dehydratase	8	18
OprD	PA0958	Porin D	11	29

BamD	PA4545	Outer membrane protein assembly factor BamD	7	20
OprF	PA1777	Outer membrane porin F	3	10
AccC	PA4848	Biotin carboxylase	6	21
Ssb	PA4232	Single-stranded DNA-binding protein	2	14
PilJ	PA0411	Protein PilJ	11	24
PhhC	PA0870	Aromatic-amino-acid aminotransferase	6	19
PilG	PA0408	Protein PilG	2	18
FtsA	PA4408	Cell division protein FtsA	4	13
FtsZ	PA4407	Cell division protein FtsZ	6	20
TolQ	PA0969	Tol-Pal system protein TolQ	2	11
TolR	PA0970	Tol-Pal system protein TolR	4	37
TolB	PA0972	Tol-Pal system protein TolB	9	27
MexB	PA0426	Multidrug resistance protein MexB	14	19
MexA	PA0425	Multidrug resistance protein MexA	11	36
SodB	PA4366	Superoxide dismutase [Fe]	2	14
AspC	PA3139	Aspartate aminotransferase	3	10
HemN	PA1546	Oxygen-independent coproporphyrinogen III oxidase	4	10
XcpT	PA3101	Type II secretion system core protein G	2	16
AtpF	PA5558	ATP synthase subunit b	9	44
DnaK	PA4761	Chaperone protein DnaK	28	59
IleS	PA4560	Isoleucine--tRNA ligase	10	13
AlaS	PA0903	Alanine--tRNA ligase	9	13
PpsA	PA1770	Phosphoenolpyruvate synthase	10	16
Icd	PA2623	Isocitrate dehydrogenase [NADP]	12	31
NuoC	PA2639	NADH-quinone oxidoreductase subunit C/D	11	21
Tal	PA2796	Transaldolase	4	16
NqrA	PA2999	Na(+)-translocating NADH-quinone reductase subunit A	8	23
GltX	PA3134	Glutamate--tRNA ligase	6	12
LysS	PA3700	Lysine--tRNA ligase	11	28
RpoC	PA4269	DNA-directed RNA polymerase subunit beta'	33	27
SpuD	PA0300	Putrescine-binding periplasmic protein SpuD	7	25
AhpC	PA0139	Alkyl hydroperoxide reductase C	5	33
Fur	PA4764	Ferric uptake regulation protein	3	27

MrcA	PA5045	Penicillin-binding protein 1A	10	14
PurF	PA3108	Amidophosphoribosyltransferase	5	11
NirF	PA0516	Protein NirF	5	15
OprB	PA3186	Porin B	15	41
OprM	PA0427	Outer membrane protein OprM	19	48
SucD	PA1589	Succinate--CoA ligase [ADP-forming] subunit alpha	10	39
AceE	PA5015	Pyruvate dehydrogenase E1 component	28	31
AceF	PA5016	Dihydrolipoamide acetyltransferase component of pyuruvate dehydrogenase complex	13	33
HemB	PA5243	Delta-aminolevulinic acid dehydratase	6	21
Rho	PA5239	Transcription termination factor Rho	6	19
FabY	PA5174	Beta-ketoacyl-[acyl-carrier-protein] synthase FabY	5	12
GpmI	PA5131	2,3-bisphosphoglycerate-independent phosphoglycerate mutase	6	15
GlnA	PA5119	Glutamine synthetase	8	19
PurD	PA4855	Phosphoribosylamine--glycine ligase	7	23
Prs	PA4670	Ribose-phosphate pyrophosphokinase	7	32
PagL	PA4661	Lipid A deacylase PagL	5	33
GlyA2	PA4602	Serine hydroxymethyltransferase 3	5	15
ClpB	PA4542	Chaperone protein ClpB	15	23
FusA	PA4266	Elongation factor G 1	26	49
PA3922	PA3922	Uncharacterized protein PA3922	11	33
SecD	PA3821	Protein translocase subunit SecD	7	12
BamB	PA3800	Outer membrane protein assembly factor BamB	8	23
RpsA	PA3162	30S ribosomal protein S1	13	31
GdhB	PA3068	NAD-specific glutamate dehydrogenase	26	19
PA2953	PA2953	Electron transfer flavoprotein-ubiquinone oxidoreductase	9	22
EtfB	PA2952	Electron transfer flavoprotein subunit beta	3	18
PA2652	PA2652	Methyl-accepting chemotaxis protein PA2652	4	12

PurB	PA2629	Adenylosuccinate lyase	6	16
ClpP1	PA1801	ATP-dependent Clp protease proteolytic subunit 1	2	15
AcnB	PA1787	Aconitate hydratase B	23	34
PA1618	PA1618	Putative esterase PA1618	2	17
HtpG	PA1596	Chaperone protein HtpG	15	30
LpdG	PA1587	Dihydrolipoyl dehydrogenase	15	42
SucB	PA1586	Dihydrolipoamide succinyltransferase (E2 subunit)	13	41
CcmH	PA1482	Cytochrome c-type biogenesis protein CcmH	2	14
AnsB	PA1337	Glutaminase-asparaginase	6	27
Pal	PA0973	Peptidoglycan-associated lipoprotein	3	23
AcsA1	PA0887	Acetyl-coenzyme A synthetase 1	7	15
MliC	PA0867	Membrane-bound lysozyme inhibitor of C-type lysozyme	5	44
Hpd	PA0865	4-hydroxyphenylpyruvate dioxygenase	15	47
FumC1	PA0854	Fumarate hydratase class II 1	3	11
CalB	PA0366	Probable coniferyl aldehyde dehydrogenase	9	25.0
DavT	PA0266	5-aminovalerate aminotransferase DavT	4	13
Hcp1	PA0085	Protein Hcp1	3	21
GyrB	PA0004	DNA gyrase subunit B	8	11
DnaN	PA0002	Beta sliding clamp	6	20
TrxA	PA5240	Thioredoxin	2	21
WbjC	-	UDP-2-acetamido-2,6-beta-L-arabino-hexul-4-ose reductase	5	18
PilY1	PA4554	Type IV pilus biogenesis factor PilY1	11	12

S2 Table: **Proteins identified from axial ablations on transwell membrane insert grown biofilms.** A comprehensive list of statistically significant *P. aeruginosa* proteins identified from three-day biofilms grown on transwell cell culture plate inserts. Proteins with blue fonts correspond to those that are higher abundance in the anoxic region compared to the oxic region, while proteins in red fonts are lower abundance in the anoxic region. The proteins without highlights are not statistically significant but meet the selection criteria (at least two unique peptides and ≥10% sequence coverage). The tabulated proteins are also portrayed on the volcano plot in Fig 6.

Protein	KEGG ID	Description of Protein	Unique Peptides	Sequence Coverage (%)	$\log_2$ ($X_{Anaerobic}$/$X_{Aerobic}$)	q-value	$-\log_{10}$ (p-value)
Icd	PA2623	Isocitrate dehydrogenase [NADP]	26	62.7	4.09	0.18	0.84
BraC	PA1074	Leucine-, isoleucine-, valine-, threonine-, and alanine-binding protein	18	70.0	3.87	0.08	1.37
SucD	PA1589	Succinate--CoA ligase [ADP-forming] subunit alpha	13	61.7	3.85	0.08	1.32
LpdG	PA1587	Dihydrolipoyl dehydrogenase	21	60.5	3.65	0.11	1.09
AhpC	PA0139	Alkyl hydroperoxide reductase C	11	73.3	3.61	0.10	1.15
RplY	PA4671	50S ribosomal protein L25	12	79.9	3.58	0.00	3.36
RplV	PA4258	50S ribosomal protein L22	6	48.2	3.33	0.01	1.86
FusA	PA4266	Elongation factor G 1	34	73.5	3.16	0.16	0.94
RpsA	PA3162	30S ribosomal protein S1	26	47.4	3.12	0.10	1.18
AnsB	PA1337	Glutaminase-asparaginase	12	51.7	3.09	0.00	3.29
HupB	PA1804	DNA-binding protein HU-beta	5	63.3	3.01	0.02	2.11
CarA	PA4758	Carbamoyl-phosphate synthase small chain	6	28.6	2.96	0.94	0.00
ArcA	PA5171	Arginine deiminase	23	66.3	2.96	0.02	1.97
EtfB	PA2952	Electron transfer flavoprotein subunit beta	12	67.1	2.85	0.11	1.09
SodB	PA4366	Superoxide dismutase [Fe]	6	40.9	2.76	0.92	0.00
PyrC'	PA0401	Dihydroorotase-like protein	6	19.6	2.65	0.85	0.00

AtpB	PA5560	ATP synthase subunit a	3	15.9	2.63	0.09	1.19
PurM	PA0945	Phosphoribosylformylglycinamidine cyclo-ligase	12	53.5	2.63	1.00	0.00
Tsf	PA3655	Elongation factor Ts	18	68.2	2.56	0.39	0.62
Gor	PA2025	Glutathione reductase	8	26.6	2.56	0.98	0.00
Psd	PA4957	Phosphatidylserine decarboxylase proenzyme	5	23.2	2.55	1.00	0.00
Prs	PA4670	Ribose-phosphate pyrophosphokinase	13	57.2	2.53	0.01	1.89
AtpD	PA5554	ATP synthase subunit beta	22	69	2.53	0.04	1.53
MscL	PA4614	Large-conductance mechanosensitive channel	3	28.5	2.46	0.00	2.63
ArgJ	PA4402	Arginine biosynthesis bifunctional protein ArgJ	9	38.3	2.45	0.00	3.97
YidC	PA5568	Membrane protein insertase YidC	11	27.7	2.45	0.04	1.66
RapA	PA3308	RNA polymerase-associated protein RapA	9	13.5	2.42	0.79	0.00
SpuD	PA0300	Putrescine-binding periplasmic protein SpuD	9	31.9	2.40	0.01	2.48
FliC	PA1092	B-type flagellin	14	44.4	2.40	0.29	0.71
ArgC	PA0662	N-acetyl-gamma-glutamyl-phosphate reductase	8	32.3	2.40	0.00	3.74
Dxs	PA4044	1-deoxy-D-xylulose-5-phosphate synthase	6	15.8	2.39	1.00	0.00
ArgG	PA3525	Argininosuccinate synthase	12	37.5	2.36	0.00	3.06
Hcp1	PA0085	Protein Hcp1	6	46.9	2.35	0.73	0.00
FusB	PA2071	Elongation factor G 2	13	40.7	2.32	0.77	0.00
PagL	PA4661	Lipid A deacylase PagL	6	45.1	2.32	0.05	1.58
RplA	PA4273	50S ribosomal protein L1	12	57.6	2.32	0.18	0.85
AtpG	PA5555	ATP synthase gamma chain	12	53.8	2.29	0.00	2.69
AtpA	PA5556	ATP synthase subunit alpha	5	43.8	2.26	0.16	0.94

Ppk2	PA0141	Polyphosphate:ADP/GDP phosphotransferase	12	46.5	2.23	0.74	0.00
AstB	PA0899	N-succinylarginine dihydrolase	8	22.8	2.17	1.00	0.00
AruC	PA0895	Succinylornithine transaminase/acetylornithine aminotransferase	17	60.3	2.12	0.01	2.44
FptA	PA4221	Fe(3+)-pyochelin receptor	25	53.2	2.12	0.13	1.06
FumC1	PA0854	Fumarate hydratase class II 1	11	40.3	2.04	0.75	0.00
GdhB	PA3068	NAD-specific glutamate dehydrogenase	50	39.1	2.01	0.03	1.73
CysS	PA1795	Cysteine--tRNA ligase	12	31.3	2.00	1.00	0.00
SpuE	PA0301	Spermidine-binding periplasmic protein SpuE	7	25.5	2.00	0.74	0.00
HemL	PA3977	Glutamate-1-semialdehyde 2,1-aminomutase	9	32.1	1.99	1.00	0.00
GalU	PA2023	UTP--glucose-1-phosphate uridylyltransferase	8	43.4	1.95	0.76	0.00
DavD	PA0265	Glutarate-semialdehyde dehydrogenase	13	39.8	1.91	0.02	2.33
PurA	PA4938	Adenylosuccinate synthetase	16	48.8	1.89	0.02	2.19
RplJ	PA4272	50S ribosomal protein L10	8	48.8	1.88	0.18	0.84
UbiG	PA3171	Ubiquinone biosynthesis O-methyltransferase	6	39.7	1.88	0.87	0.00
GyrB	PA0004	DNA gyrase subunit B	20	35.5	1.87	0.73	0.00
IleS	PA4560	Isoleucine--tRNA ligase	16	24.1	1.87	0.89	0.00
PurH	PA4854	Bifunctional purine biosynthesis protein PurH	11	27.5	1.86	0.08	1.35
Pnp	PA4740	Polyribonucleotide nucleotidyltransferase	26	50.8	1.85	0.08	1.31
Pal	PA0973	Peptidoglycan-associated lipoprotein	7	66.7	1.84	0.34	0.67
OprB	PA3186	Porin B	21	58.1	1.84	0.49	0.55

PA3286	PA3286	Beta-ketodecanoyl-[acyl-carrier-protein] synthase	8	37.1	1.83	0.78	0.00
DnaK	PA4761	Chaperone protein DnaK	26	63.9	1.81	0.50	0.54
TufA; TufB	PA4265	Elongation factor Tu	21	77.3	1.81	0.21	0.80
Hpd	PA0865	4-hydroxyphenylpyruvate dioxygenase	12	45.9	1.80	0.00	2.70
PA2953	PA2953	Electron transfer flavoprotein-ubiquinone oxidoreductase	19	46.5	1.80	0.03	1.75
RplP	PA4256	50S ribosomal protein L16	5	40.1	1.79	0.01	2.44
MetZ	PA3107	O-succinylhomoserine sulfhydrylase	7	23.8	1.79	0.91	0.00
ArgB	PA5323	Acetylglutamate kinase	5	20.9	1.77	1.00	0.00
FumC2	PA4470	Fumarate hydratase class II 2	11	32.5	1.77	0.86	0.00
MexB	PA0426	Multidrug resistance protein MexB	14	18.4	1.73	0.01	2.56
MetE	PA1927	5-methyltetrahydropteroyltriglutamate-homocysteine methyltransferase	18	27.8	1.68	0.02	2.06
PepA	PA3831	Cytosol aminopeptidase	19	50.9	1.66	0.02	2.00
Cmk	PA3163	Cytidylate kinase	4	25.3	1.62	1.00	0.00
GyrA	PA3168	DNA gyrase subunit A	14	20.8	1.62	0.00	3.69
MmsB	PA3569	3-hydroxyisobutyrate dehydrogenase	5	20.1	1.60	0.96	0.00
ProS	PA0956	Proline--tRNA ligase	15	38.7	1.58	0.05	1.56
GlmS	PA5549	Glutamine--fructose-6-phosphate aminotransferase [isomerizing]	12	30.4	1.57	0.84	0.00
SecG	PA4747	Protein-export membrane protein SecG	3	37.2	1.55	0.82	0.00
MaeA	PA3471	NAD-dependent malic enzyme	10	30.9	1.54	0.87	0.00

Anr	PA1544	Transcriptional activator protein anr	3	16.4	1.51	0.97	0.00
WbjC	-	UDP-2-acetamido-2,6-beta-L-arabino-hexul-4-ose reductase	7	29.8	1.51	0.72	0.00
Eda	PA3181	2-dehydro-3-deoxy-phosphogluconate aldolase	5	26.4	1.49	0.08	1.39
AroB	PA5038	3-dehydroquinate synthase	4	17.7	1.45	1.00	0.00
MmsA	PA3570	Methylmalonate-semialdehyde dehydrogenase [acylating]	12	34.6	1.44	0.96	0.00
OprD	PA0958	Porin D	13	37.7	1.42	0.84	0.38
AlgC	PA5322	Phosphomannomutase/ phosphoglucomutase	13	35.6	1.42	0.00	3.08
AguA	PA0292	Agmatine deiminase	3	13.6	1.41	1.00	0.00
Eco	PA2755	Ecotin	5	41.7	1.41	1.00	0.00
TyrS2	PA0668	Tyrosine--tRNA ligase 2	10	29.3	1.39	0.74	0.00
AcsA1	PA0887	Acetyl-coenzyme A synthetase 1	23	55	1.39	0.22	0.78
HisA	PA5141	1-(5-phosphoribosyl)-5-[(5-phosphoribosylamino) methylideneamino] imidazole-4-carboxamide isomerase	5	25.7	1.39	0.13	1.06
ValS	PA3834	Valine--tRNA ligase	24	30.8	1.38	0.18	0.83
SucC	PA1588	Succinate--CoA ligase [ADP-forming] subunit beta	13	62.1	1.37	0.69	0.45
Efp	PA2851	Elongation factor P	6	33.5	1.35	0.02	2.17
RplQ	PA4237	50S ribosomal protein L17	5	49.6	1.34	1.00	0.00
ClpB	PA4542	Chaperone protein ClpB	35	55.4	1.33	0.03	1.78
MurD	PA4414	UDP-N-acetylmuramoylalanine--D-glutamate ligase	5	12.3	1.31	0.80	0.00
AccD	PA3112	Acetyl-coenzyme A carboxylase carboxyl transferase subunit beta	4	29.7	1.31	0.88	0.00

BamD	PA4545	Outer membrane protein assembly factor BamD	13	49	1.26	0.08	1.28
PA1579	PA1579	Uncharacterized protein PA1579	5	26.7	1.26	0.02	1.89
Pgk	PA0552	Phosphoglycerate kinase	15	51.7	1.25	0.03	1.71
AccC	PA4848	Biotin carboxylase	14	45.4	1.25	0.00	3.65
Def	PA0019	Peptide deformylase	3	21.4	1.24	0.73	0.00
DavT	PA0266	5-aminovalerate aminotransferase DavT	14	52.1	1.24	0.08	1.35
HtpG	PA1596	Chaperone protein HtpG	36	66.6	1.22	1.00	0.28
HisG	PA4449	ATP phosphoribosyltransfer ase	5	34.1	1.22	1.00	0.00
MetG	PA3482	Methionine--tRNA ligase	21	40.4	1.20	0.40	0.61
BamB	PA3800	Outer membrane protein assembly factor BamB	10	41.8	1.20	0.79	0.00
Qor	PA0023	Quinone oxidoreductase	10	42.2	1.20	0.17	0.87
AcsA2	PA4733	Acetyl-coenzyme A synthetase 2	11	29.3	1.19	0.40	0.62
SerC	PA3167	Phosphoserine aminotransferase	11	35.7	1.19	1.00	0.00
ThiG	PA0381	Thiazole synthase	4	22.3	1.18	1.00	0.00
SthA	PA2991	Soluble pyridine nucleotide transhydrogenase	14	39.9	1.18	0.03	1.71
PA4395	PA4395	UPF0234 protein PA4395	11	50.9	1.16	0.08	1.29
FimW	PA4958	Cyclic-di-GMP receptor FimW	5	11.7	1.16	0.83	0.00
PurL	PA3763	Phosphoribosylformylg lycinamidine synthase	16	17.5	1.15	0.79	0.00
SurA	PA0594	Chaperone SurA	10	29.3	1.14	0.10	1.16
ArgS	PA5051	Arginine--tRNA ligase	2	27.8	1.14	0.02	2.02
BkdB	PA2249	Lipoamide acyltransferase component of branched-chain alpha-keto acid dehydrogenase complex	6	15.9	1.12	0.77	0.00

ArcB	PA5172	Ornithine carbamoyltransferase, catabolic	20	68.5	1.11	0.02	1.79
PckA	PA5192	Phosphoenolpyruvate carboxykinase (ATP)	2	49.9	1.08	0.05	1.63
ZipA	PA1528	Cell division protein ZipA	5	25.3	1.06	1.00	0.00
FabZ	PA3645	3-hydroxyacyl-[acyl-carrier-protein] dehydratase FabZ	4	37.7	1.03	1.00	0.00
FabG	PA2967	3-oxoacyl-[acyl-carrier-protein] reductase FabG	7	40.1	1.03	0.00	2.88
Mqo2	PA4640	Probable malate:quinone oxidoreductase 2	8	18.5	1.02	0.82	0.00
PyrE	PA5331	Orotate phosphori-bosyltransferase	6	35.7	1.01	1.00	0.00
AhcY	PA0432	Adenosylhomocysteinase	20	56.5	1.01	0.02	1.94
AtoB	PA2001	Acetyl-CoA acetyltransferase	8	32.8	0.98	0.76	0.00
PyrG	PA3637	CTP synthase	22	53	0.96	0.11	1.11
ExaA	PA1982	Quinoprotein ethanol dehydrogenase (cytochrome c)	19	40.9	0.94	0.16	0.90
Edd	PA3194	Phosphogluconate dehydratase	15	39.5	0.93	0.01	2.50
LysS	PA3700	Lysine--tRNA ligase	23	49.1	0.89	0.02	2.09
RplU	PA4568	50S ribosomal protein L21	7	67	0.89	0.67	0.47
PilY1	PA4554	Type IV pilus biogenesis factor PilY1	20	25.4	0.86	0.02	1.93
Fmt	PA0018	Methionyl-tRNA formyltransferase	5	25.8	0.85	0.86	0.00
Pgl	PA3182	6-phosphogluconolactonase	5	29.4	0.82	0.08	1.26
PurC	PA1013	Phosphoribosylaminoimidazole-succinocarboxamide synthase	8	41.1	0.82	0.20	0.81
ThrC	PA3735	Threonine synthase	11	31.3	0.82	0.96	0.00
LepA	PA0767	Elongation factor 4	6	12.5	0.80	1.00	0.00
Ggt	PA1338	Glutathione hydrolase proenzyme	8	20.3	0.79	0.75	0.00

NuoB	PA2638	NADH-quinone oxidoreductase subunit B	3	18.7	0.78	1.00	0.00
GlnS	PA1794	Glutamine--tRNA ligase	13	31.7	0.78	0.08	1.27
CheB1	PA1459	Protein-glutamate methylesterase/protein-glutamine glutaminase 1	6	25	0.76	1.00	0.00
RpsD	PA4239	30S ribosomal protein S4	13	48.5	0.75	0.05	1.61
ArgH	PA5263	Argininosuccinate lyase	10	29.1	0.74	0.92	0.00
PheA	PA3166	Bifunctional chorismate mutase/prephenate dehydratase	6	21.9	0.73	0.78	0.00
Glk	PA3193	Glucokinase	7	32.6	0.73	0.87	0.00
MexA	PA0425	Multidrug resistance protein MexA	12	52.2	0.72	0.05	0.20
GroEL	PA4385	60 kDa chaperonin	19	55.9	0.71	1.00	0.00
KdsA	PA3636	2-dehydro-3-deoxyphosphooctonate aldolase	7	25.3	0.71	1.00	0.69
RpsP	PA3745	30S ribosomal protein S16	3	48.2	0.69	0.31	0.99
PpsA	PA1770	Phosphoenolpyruvate synthase	32	51.2	0.68	0.14	0.00
SpeE2	PA4774	Polyamine aminopropyltransferase 2	6	25.5	0.68	0.82	0.00
RdgC	PA3263	Recombination-associated protein RdgC	11	50.7	0.67	1.00	0.00
SpuC	PA0299	Putrescine--pyruvate aminotransferase	9	35.1	0.66	0.74	0.00
OprF	PA1777	Outer membrane porin F	18	67.1	0.65	1.00	0.18
GlyS	PA0008	Glycine--tRNA ligase beta subunit	14	28.9	0.65	0.10	1.16
RibB	PA4054	3,4-dihydroxy-2-butanone 4-phosphate synthase	7	27.7	0.64	0.98	0.31
PanB2	PA4729	3-methyl-2-oxobutanoate hydroxymethyltransferase 2	3	14.3	0.63	0.89	0.00

GlnA	PA5119	Glutamine synthetase	17	44.6	0.62	0.92	0.33
CalB	PA0366	Probable coniferyl aldehyde dehydrogenase	13	37.8	0.62	0.04	1.64
ProA	PA4007	Gamma-glutamyl phosphate reductase	10	30.9	0.62	1.00	0.00
PctB	PA4310	Methyl-accepting chemotaxis protein PctB	3	29.7	0.62	0.80	0.00
TrpS	PA4439	Tryptophan--tRNA ligase	5	17.4	0.60	0.80	0.00
NusG	PA4275	Transcription termination/antitermination protein NusG	5	35	0.60	0.02	1.98
GuaA	PA3769	GMP synthase [glutamine-hydrolyzing]	22	55.8	0.59	0.00	2.76
TrpE	PA0609	Anthranilate synthase component 1	11	32.1	0.58	0.99	0.00
RpoB	PA4270	DNA-directed RNA polymerase subunit beta	64	54.9	0.58	1.00	0.25
PilJ	PA0411	Protein PilJ	15	30.2	-0.58	0.08	1.29
PctA	PA4309	Methyl-accepting chemotaxis protein PctA	7	37.8	-0.61	0.62	0.49
RpsF	PA4935	30S ribosomal protein S6	4	25.9	-0.62	1.00	0.00
RpsQ	PA4254	30S ribosomal protein S17	4	42	-0.62	0.18	0.83
AceF	PA5016	Dihydrolipoyllysine-residue acetyltransferase component of pyruvate dehydrogenase complex	22	48.6	-0.63	0.15	0.94
InfC	PA2743	Translation initiation factor IF-3	8	55.2	-0.64	0.43	0.59
AspS	PA0963	Aspartate--tRNA(Asp/Asn) ligase	12	38.4	-0.67	0.02	2.25
BraF	PA1071	High-affinity branched-chain amino acid transport ATP-binding protein BraF	8	41.2	-0.67	0.98	0.00
PA3647	PA3647	Skp-like protein	3	24.4	-0.67	0.79	0.00

LpxC	PA4406	UDP-3-O-acyl-N-acetylglucosamine deacetylase	11	40.3	-0.67	0.05	1.51
MurC	PA4411	UDP-N-acetylmuramate--L-alanine ligase	5	14.4	-0.69	1.00	0.00
IlvD	PA0353	Dihydroxy-acid dehydratase	12	31.7	-0.71	1.00	0.00
HldE	PA4996	Bifunctional protein HldE	6	15.9	-0.71	1.00	0.00
Hfq	PA4944	RNA-binding protein Hfq	6	75.6	-0.71	1.00	0.00
Ndk	PA3807	Nucleoside diphosphate kinase	6	49	-0.71	0.67	0.47
RpmC	PA4255	50S ribosomal protein L29	3	60.3	-0.73	1.00	0.00
RpsE	PA4246	30S ribosomal protein S5	6	50	-0.74	0.16	0.90
Fba	PA0555	Fructose-bisphosphate aldolase	12	49.2	-0.75	0.15	0.97
RpsB	PA3656	30S ribosomal protein S2	11	57.3	-0.76	0.31	0.69
AtpF	PA5558	ATP synthase subunit b	10	62.8	-0.79	0.01	1.85
AotP	PA0892	Arginine/ornithine transport ATP-binding protein AotP	5	20.9	-0.79	1.00	0.00
GapA	PA3195	Glyceraldehyde-3-phosphate dehydrogenase-like protein	16	49	-0.81	0.02	2.11
ThrS	PA2744	Threonine--tRNA ligase	21	39.5	-0.83	0.04	1.53
DnaJ	PA4760	Chaperone protein DnaJ	13	35	-0.86	1.00	0.00
PasP	PA0423	UPF0312 protein PA0423	8	55.5	-0.86	1.00	0.00
NqrF	PA2994	Na(+)-translocating NADH-quinone reductase subunit F	9	24.1	-0.89	0.88	0.00
Ppx	PA5241	Exopolyphosphatase	7	18.8	-0.90	0.72	0.00
ArcC	PA5173	Carbamate kinase	12	42.9	-0.91	0.08	1.24
Adk	PA3686	Adenylate kinase	13	59.1	-0.93	0.06	1.42
RplC	PA4263	50S ribosomal protein L3	7	35.1	-0.94	0.24	0.76

RplO	PA4244	50S ribosomal protein L15	6	45.1	-0.96	0.06	1.42
RpsG	PA4267	30S ribosomal protein S7	11	54.5	-0.97	0.16	0.91
Pgi	PA4732	Glucose-6-phosphate isomerase	9	21.8	-0.98	1.00	0.00
PheT	PA2739	Phenylalanine--tRNA ligase beta subunit	16	32.4	-1.03	0.78	0.00
Ssb	PA4232	Single-stranded DNA-binding protein	8	63	-1.05	0.14	0.99
ClpX	PA1802	ATP-dependent Clp protease ATP-binding subunit ClpX	12	35	-1.08	1.00	0.00
RpsH	PA4249	30S ribosomal protein S8	7	50.8	-1.08	0.01	1.89
RpsJ	PA4264	30S ribosomal protein S10	3	34	-1.09	0.06	1.42
GroES	PA4386	10 kDa chaperonin	1	17.5	-1.09	1.00	0.00
RplN	PA4253	50S ribosomal protein L14	6	47.5	-1.10	1.00	0.00
PilQ	PA5040	Fimbrial assembly protein PilQ	12	22.1	-1.11	0.30	0.69
EstA	PA5112	Esterase EstA	9	15.9	-1.11	1.00	0.00
RpsM	PA4241	30S ribosomal protein S13	8	65.3	-1.13	0.05	1.55
BkdA1	PA2247	2-oxoisovalerate dehydrogenase subunit alpha	5	16.8	-1.15	0.77	0.00
AtpH	PA5557	ATP synthase subunit delta	5	27.5	-1.16	0.17	0.89
GltX	PA3134	Glutamate--tRNA ligase	6	19.8	-1.22	0.88	0.00
RplB	PA4260	50S ribosomal protein L2	14	53.5	-1.22	0.14	0.99
TatA	PA5068	Sec-independent protein translocase protein TatA	3	53.7	-1.26	1.00	0.00
PheS	PA2740	Phenylalanine--tRNA ligase alpha subunit	12	34.9	-1.29	0.89	0.00
TpiA	PA4748	Triosephosphate isomerase	5	45.8	-1.34	1.00	0.00
LysA	PA5277	Diaminopimelate decarboxylase	8	26.5	-1.34	0.99	0.00
GlpK2	PA3582	Glycerol kinase 2	10	22.6	-1.36	0.05	1.51

HmgA	PA2009	Homogentisate 1,2-dioxygenase	7	20.4	-1.37	1.00	0.00
SecY	PA4243	Protein translocase subunit SecY	5	13.6	-1.39	0.80	0.00
Frr	PA3653	Ribosome-recycling factor	7	42.7	-1.39	1.00	0.00
ClpV1	PA0090	AAA + ATPase ClpV1	8	13	-1.41	0.73	0.00
RpsL	PA4268	30S ribosomal protein S12	4	43.9	-1.42	1.00	0.00
LeuA	PA3792	2-isopropylmalate synthase	13	39.7	-1.46	1.00	0.00
Gap	PA3195	Glyceraldehyde-3-phosphate dehydrogenase	14	55.7	-1.55	0.03	1.71
PurD	PA4855	Phosphoribosylamine--glycine ligase	6	20.3	-1.58	0.82	0.00
GlyA2	PA4602	Serine hydroxymethyltransferase 3	12	45.8	-1.59	0.05	1.44
RpmG	PA5315	50S ribosomal protein L33	2	43.1	-1.64	0.04	1.54
RplR	PA4247	50S ribosomal protein L18	7	51.7	-1.64	0.05	1.51
RpsK	PA4240	30S ribosomal protein S11	4	41.9	-1.67	0.02	1.79
MrcA	PA5045	Penicillin-binding protein 1A	9	12.7	-1.72	0.86	0.00
Tpx	PA2532	Thiol peroxidase	6	55.8	-1.74	0.91	0.00
RplM	PA4433	50S ribosomal protein L13	5	54.2	-1.79	0.10	1.14
RplT	PA2741	50S ribosomal protein L20	3	22.9	-1.80	1.00	0.00
AceA	PA2634	Isocitrate lyase	11	24.5	-1.84	0.04	1.66
RpsS	PA4259	30S ribosomal protein S19	3	28.6	-1.86	0.04	1.61
PrfA	PA4665	Peptide chain release factor 1	3	11.9	-1.89	1.00	0.00
SelD	PA1642	Selenide, water dikinase	4	16.6	-1.90	0.76	0.00
DnaK	PA4761	Chaperone protein DnaK	3	19	-1.92	0.06	1.40
Pta	PA0835	Phosphate acetyltransferase	9	20.2	-1.93	0.75	0.00

UbiE	PA5063	Ubiquinone/menaquinone biosynthesis C-methyltransferase UbiE	10	49.2	-1.94	1.00	0.00
GcvP1	PA2445	Glycine dehydrogenase (decarboxylating) 1	10	17.7	-1.94	0.78	0.00
HemN	PA1546	Oxygen-independent coproporphyrinogen III oxidase	7	22.2	-1.98	0.91	0.00
ArgF	PA3537	Ornithine carbamoyltransferase, anabolic	5	23	-2.21	1.00	0.00
RpmD	PA4245	50S ribosomal protein L30	2	25.9	-2.26	1.00	0.00
RplL	PA4271	50S ribosomal protein L7/L12	3	19.7	-2.84	1.00	0.00
