# Supplementary figures and images for: Spatially resolved analysis of Pseudomonas aeruginosa biofilm proteomes measured by laser ablation sample transfer
Source: PLoS One. 2021 Jul 22;16(7):e0250911. doi: 10.1371/journal.pone.0250911 (PMC8297752; doi:10.1371/journal.pone.0250911)

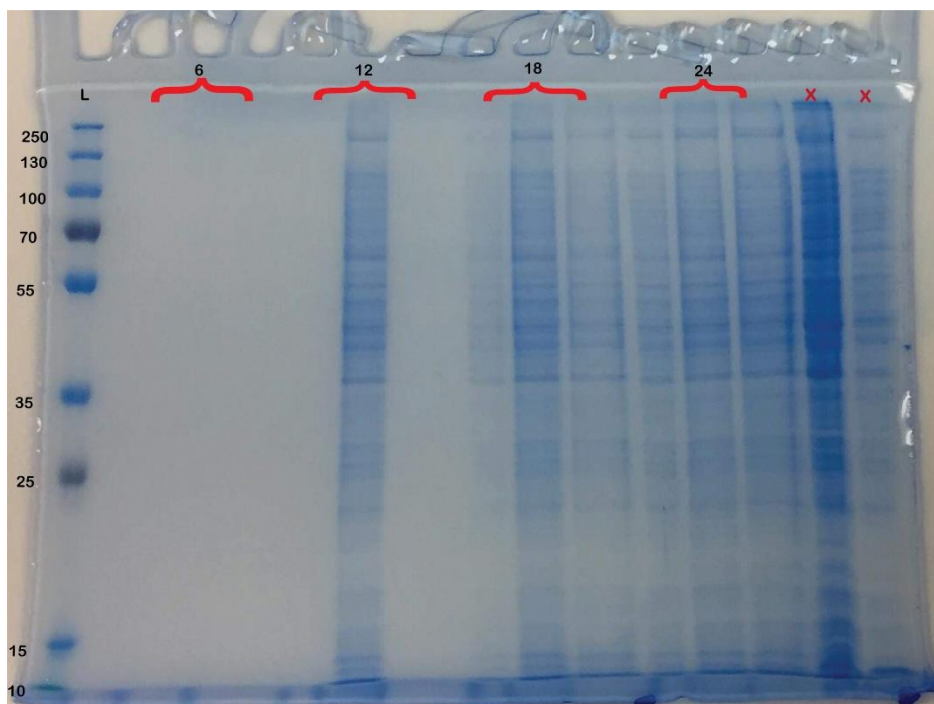

Raw gel for Figure 3a

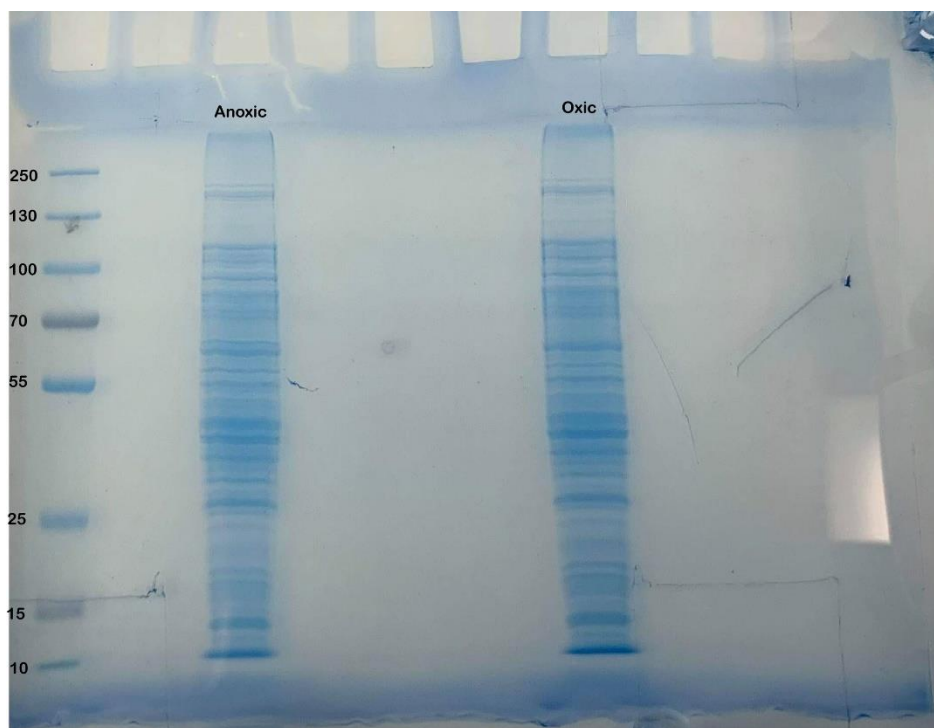

Raw gel for Figure 3b

Supplement: S1 Raw images — These are the unprocessed gel images from SDS-PAGE. (PDF) [file pone.0250911.s002.pdf]

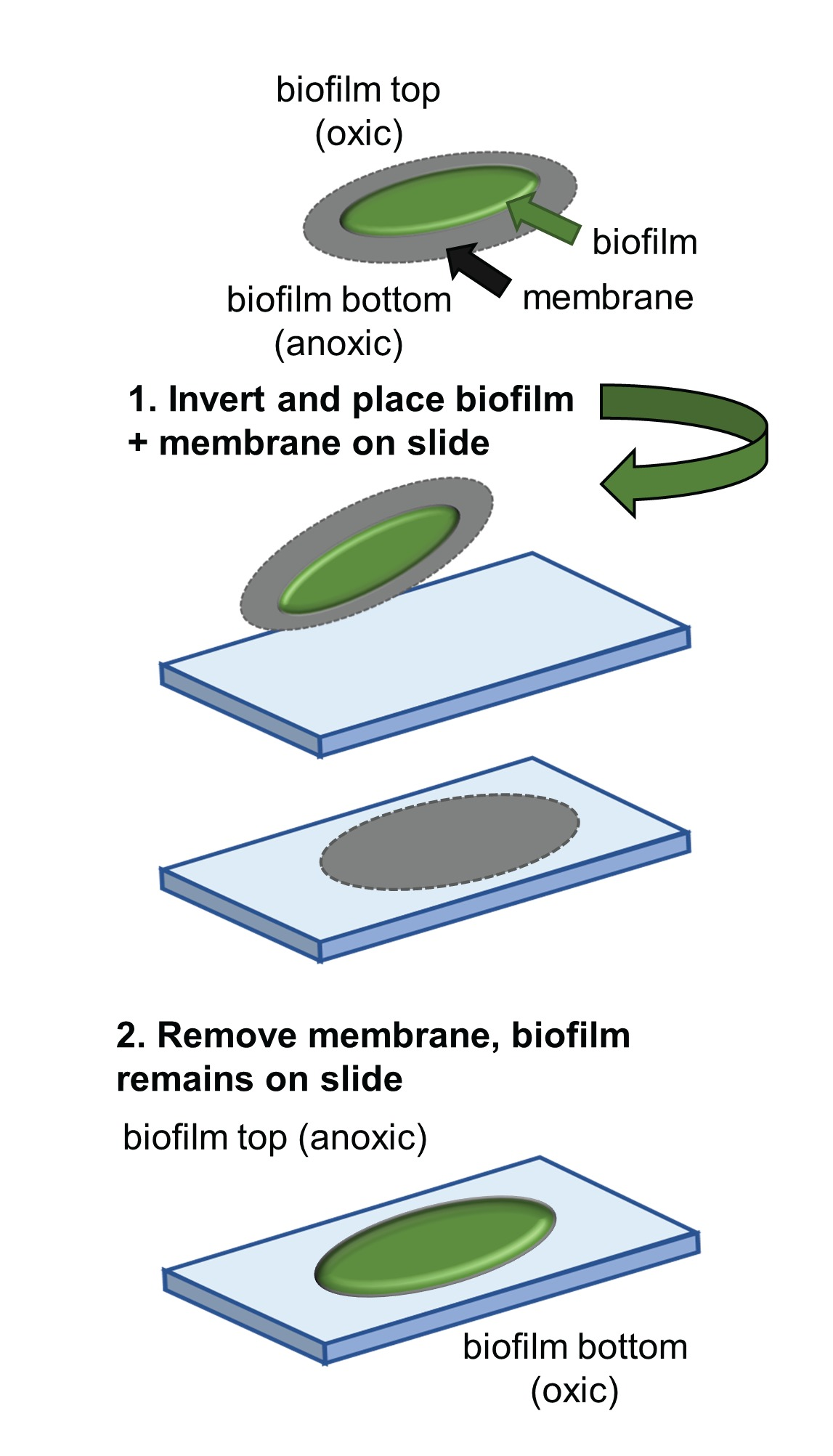

Supplement: S1 Fig — (TIF) [file pone.0250911.s003.tif]
