# Supplementary material for: Spatially resolved analysis of Pseudomonas aeruginosa biofilm proteomes measured by laser ablation sample transfer
Source: PLoS One. 2021 Jul 22;16(7):e0250911. doi: 10.1371/journal.pone.0250911 (PMC8297752; doi:10.1371/journal.pone.0250911)
Supplement: Attachment — Submitted filename: Response to Reviewers - PONE-D-21-11668R1.pdf [file pone.0250911.s004.pdf]

UIC UNIVERSITY OF ILLINOIS AT CHICAGO

Department of Chemistry (MC 111), Science & Engineering South
845 West Taylor Street, Room 4500, Chicago, Illinois 60607-4036
Office: 1-312-996-0945, Fax: (312) 996-0431, Email: LHanley@uic.edu

June 14, 2021

To Thierry Rabilloud, Academic Editor, PLOS ONE

I am writing to resubmit our manuscript **PONE-D-21-11668** to your journal:

Spatially resolved analysis of *Pseudomonas aeruginosa* biofilm proteomes measured by laser ablation sample transfer

by Aruni Chathurya Pulukkody, Yeni P. Yung, Fabrizio Donnarumma, Kermit K. Murray, Ross P. Carlson, and Luke Hanley

We thank you in advance for your reconsideration of this manuscript. We have uploaded all of the requested files, including this file as the

- rebuttal letter (PONE-D21-11668 Rebuttal Lett.pdf),
- marked-up copy of the manuscript with changes highlighted (PONE-D-21-11668 Marked Manuscript Revised.pdf),
- unmarked plain text manuscript (PONE-D21-11668 Manuscript Plain Text.docx), and
- revised Supporting Information (S1_File.pdf).

The changes to the manuscript are summarized below, in the order they were raised by your office or the reviewers but using our own sequential numbering scheme.

Responses to Comments by Editorial Office

1. We have revised the author affiliations and reformatted the manuscript to adhere to the journal guidelines.
2. In response to the request that we include details of the source of the bacterial cell line, we have cited the reference that first refers to this clinical strain.
3. We have upload the raw gel images as a supporting file "S1_raw_images.pdf", as directed in the journal guidelines.

Responses to Reviewer #1

4. We have made the data that we uploaded onto the MassIVe repository public. It was our oversight to not have done this upon the initial submission.
5. See point 2. above regarding the request for information on bacterial strain. This strain has unfortunately not been sequence, to our knowledge.
6. Regarding whether ion intensities or LFW were used for quantification of proteins, the text has been changed in the 2nd paragraph of p. 9 to clarify that ion intensities were converted by the Perseus software into a semi-quantitative measure of protein abundances.
7. 3696 protein entries were searched in UniProt, as now noted in the 2nd paragraph of p. 9.
8. FDR are not calculated by the user of MaxQuant or Perseus. We selected standard FDR values of 0.01 for MaxQuant and 0.05 for Perseus, as originally noted (no changes to manuscript).
9. Regarding the question of replicates, we note that we sampled three biological replicates for each of the oxic and anoxic regions of the transwell membrane biofilms, each leading to three separate analyses by LC-MS/MS. However, we note that samples from six polycarbonate biofilms were pooled for a single LC-MS/MS analysis of the old and new regions, for which no statistics were provided. See last paragraph of p. 9 and 1st paragraph of p. 10.

10. We were asked to discuss the original comment “This was in stark contrast to previous analyses of vertical differences in transcriptome…” See point 12. Below for our response.
11. As requested, we have added the glucose catabolizing proteins that display significance changes in abundance (Edd, Pgk, and PckA) to the volcano plot, now Fig 6.
12. The reviewer wanted us to further discuss the unexpected observation that proteins involved in glucose catabolism and substrate level phosphorylation are higher abundant in anoxic regions (this is an expansion of point 10.). We responded by consolidating and expanding our comments on these points in the 1st paragraph of p. 14 (after Fig. 6 caption) to include the new comment that “the higher abundance of proteins in the anoxic region might derive from its proximity to the growth media [10] which might drive catabolism more than oxygen abundance.” Ref. 10 has been added to support this argument.
13. We have corrected the text in the last paragraph of p. 4 to fully spell out the bacterial names upon first mention, as suggested.
14. As suggested, we have added reference to Fig 4B in the Results. However, we have done so where we consider the transwell membranes proteomics. The new sentence on the last paragraph of p. 12 reads: “Fig 4B shows SDS-PAGE of cell lysates from 24 mm^2 area regions that were laser ablated from anoxic and oxic regions of biofilms grown on transwell membranes.”
15. As suggested by the reviewer, we have dropped the terminology of upregulation/downregulation and replaced it throughout the Results and Discussion with higher and lower protein abundances. See p. 13 onward.
16. As suggested, we changed the terminology on the 2nd paragraph of p. 3. It now reads “…while metabolic activity has been measured using reporter proteins [6,9,10] and transcriptomic analyses [6,9].”
17. We added the verb “was” to the sentence discussing Fig 3. More extensively, we modified both Fig 3 and its caption to clarify the biofilm flipping procedure.
18. We deleted the following sentence that the reviewer found confusing: “The bands on each well represents the separation of proteins from the ablated material.”
19. We restructured the first sentence of p. 3 that contains the phrase “nosocomial infections” to make it clearer.
20. To address the Reviewer’s concern, we changed part of the Abstract to instead read: “This study reveals a more active metabolism in the anoxic region of the biofilm with respect to the oxic region for this clinical strain of *P. aeruginosa*, despite this organism being considered an aerobe by nature.”

Responses to Reviewer #2

21. As suggested, we moved the LAST apparatus diagram from Supp. Info. to the primary manuscript and split it to become Fig 1 and Fig 2. This led to an incrementing of the remaining figure numbers.
22. See point 4. above regarding the now public availability of the proteomic data.
23. Regarding use of Pseudomonas.com, we have changed the 2nd paragraph of p. 9 to read: “The MaxQuant computational platform [44] was used for protein identification using the UniProt Knowledgebase [45], which incorporates the data curated by Pseudomonas.com [46].” Ref. 46 has been added along with the subsequent sentence: “KEGG identifications were obtained using Pseudomonas.com if the PseudoCap identification was not found on UniProt.” We also note that MaxQuant requires files in the “FASTA” format, which are provided by UniProt, but not Pseudomonas.com.
24. As requested, we have noted in the last paragraph of p. 9 that we searched acetylation (N-terminus).
25. As implied, we have shifted to use of a factor of two or greater in protein abundance differentials. Fig 6, the associated text on p. 13 onward, and S2 Table have all been so modified.

26. The reviewer questioned the 1.3 factor in the legend of Fig 6. A volcano plot such as Fig 6 correlates statistical significance with protein abundance. A statistical significance of $p = 0.05$ corresponds to a $-\log10(p\ value) > 1.3$. No changes were made to the manuscript.
27. The reviewer requested further discussion in the final section of the Results and Discussion regarding the implications to biofilms of protein abundance changes. We partially addressed this in point 12. However, we are reluctant to make further comments on the implications of these results lest we move beyond what we can reasonably interpret from the present dataset. Future experiments are now being planned to illuminate such issues further.

We have also made many formatting changes to fit the PLOS ONE requirements and additional changes to language for the sake of clarity. All are marked on the highlighted version of the manuscript.

Thank you again for your careful consideration of our work. We look forward to your response.

Sincerely,

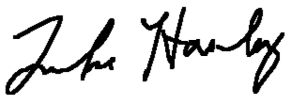

Luke Hanley,
LAS Distinguished Professor
